# Interaction of Gut Microbiota with Endocrine Homeostasis and Thyroid Cancer

**DOI:** 10.3390/cancers14112656

**Published:** 2022-05-27

**Authors:** Qi Liu, Wei Sun, Hao Zhang

**Affiliations:** Department of Thyroid Surgery, The First Hospital of China Medical University, Shenyang 110001, China; qliu87@cmu.edu.cn (Q.L.); sun19890208@126.com (W.S.)

**Keywords:** gut microbiota, thyroid cancer, Hashimoto’s thyroiditis, Graves’ disease, microbial metabolites

## Abstract

**Simple Summary:**

As the interaction between gut microbiota and thyroid cancer is gradually being revealed, the thyroid gland, as an important endocrine organ, constitutes the gut-thyroid axis. Dysbiosis of the gut microbiota leads to the accumulation of metabolites that, through specific mechanisms, lead to genetic instability in the thyroid, ultimately leading to tumorigenesis and progression. In addition, these metabolites can lead to autoimmune responses, resulting in imbalance of endocrine homeostasis and the development of autoimmune diseases. In this review, we discuss the interaction of gut microbiota with thyroid disease and thyroid cancer. We hope to provide some potential new strategies for the prevention and treatment of thyroid disease or thyroid cancer.

**Abstract:**

The gut microbiota plays a crucial role in healthy individuals as well as in patients with thyroid diseases, including thyroid cancer. Although the prognosis of differentiated thyroid cancer is predictable, that of some poorly differentiated, medullary, and anaplastic thyroid cancers remains unpromising. As the interaction between the gut microbiota and thyroid cancer has been gradually revealed in recent years, the thyroid gland, a crucial endocrine organ, is shown to have a complex connection with the body’s metabolism and is involved in inflammation, autoimmunity, or cancer progression. Dysbiosis of the gut microbiota and its metabolites can influence changes in hormone levels and susceptibility to thyroid cancer through multiple pathways. In this review, we focus on the interactions of the gut microbiota with thyroid function diseases and thyroid cancer. In addition, we also discuss some potential new strategies for the prevention and treatment of thyroid disease and thyroid cancer. Our aim is to provide some possible clinical applications of gut microbiota markers for early diagnosis, treatment, and postoperative management of thyroid cancer. These findings were used to establish a better multi-disciplinary treatment and prevention management strategy and to individualize the treatment of patients in relation to their gut microbiota composition and pathological characteristics.

## 1. Introduction

Thyroid cancer (TC), an endocrine malignancy, has plagued the world, and its incidence has been rising in recent years, leading to a wider concern worldwide [1,2]. Although the overall prognosis of differentiated thyroid cancer, such as papillary thyroid cancer (PTC), is good, that of poorly differentiated, medullary, and anaplastic thyroid cancers remains unsatisfactory [3,4]. Although external factors, such as environmental pollution and radiation exposure, are the causative factors of TC, other causative factors and prevention strategies for TC remain unclear. Originally, some organs such as the thyroid and bladder were thought to be free of bacterial colonization; however, after the development of genetic sequencing technologies, these organs were shown to be colonized by a large microbiota [5,6].

There are trillions of microorganisms colonizing the human gut, of which bacteria are the most dominant group [7]. Gut microbiota (GM) dysbiosis is a condition whereby the composition and physiological functions of the gastrointestinal microbiota are altered, thus resulting in localized enteritis and altered metabolic functions. Dysbiosis of the GM composition leading to overgrowth of some subpopulations of bacteria increases the intestinal permeability, as well as transferring pro-inflammatory cells, which potentially exert a microbial impact on the intestine. GM has an important impact on human health and the maintenance of normal physiology, such as regulating immune function, promoting the absorption of indigestible nutrients, and inhibiting pathogen colonization [8]. However, abnormalities in the composition of the intestinal flora are associated with many diseases, including metabolic and endocrine disorders [9,10]. Symbiotic GM is highly diverse, stable, resistant, and adaptable, whereas GM with dysbiosis has a low relative abundance and lacks symbiosis and diversity. The intestinal microbiota plays an important role in maintaining the nutritional, metabolic, and immune homeostasis of the host [11]. GM may be a key regulatory hub for health and disease, and the relevance of its impact on human health is now emerging. It is becoming increasingly clear that the activity of the GM, particularly their metabolites, influences the protective or promotive effects of cancers.

Microbiota may influence the secretion of the thyroid-stimulating hormone through the hypothalamus–pituitary axis, thus exerting its role in thyroid diseases [12]. The relationship between autoimmune thyroid diseases and GM has received much research attention [13,14]. Several studies have investigated the GM composition in patients with normal thyroid and hypothyroid Hashimoto’s thyroiditis (HT) and found dysbiosis or microbiota overgrowth in these patients’ gut [14,15]. The main source of influence on thyroid function is the continuous accumulation of metabolites from disordered GM [16]. HT is a relatively common thyroid disease, wherein the body produces an auto-antibody against thyroid peroxidase and thyroglobulin (Tg), thus leading to the development of chronic inflammation and often leading to the destruction of the thyroid gland [17,18]. HT is characterized by the progressive depletion of thyroid cells resulting in decreased thyroid hormone levels and clinical hypothyroidism [19]. Graves’ disease (GD) is characterized by thyroid-stimulating receptors autoantibodies that cause hyperthyroidism [20,21]. Both diseases will eventually lead to disorders of human metabolic processes. Accumulating evidence suggests the existence of a gut–endocrine–thyroid axis. The neurological, endocrine, metabolic, microbial, and immune systems together influence the composition of the GM, and GM in turn is also considered to be an important factor affecting thyroid homeostasis [22]. In addition, thyroid homeostasis may be regulated by the influence of GM on the immune system and metabolism of micronutrients.

Although cancer is the result of a combination of genetic and environmental factors, previous studies suggest that 20% of malignancies are associated with microorganisms [23]. Some bacteria and metabolites can cause genetic damage, leading to genetic instability [24]. Bacterial communities within the host may be another environmental factor associated with and contributing to TC, which until recently was not acknowledged. Several bacteria and viruses have been implicated in the development of cancers, including Helicobacter pylori, hepatitis B/C virus, and human papillomavirus [25,26]. Owing to the development of 16S rRNA gene sequencing and other technologies, various bacteria can be accurately distinguished and quantitatively analyzed [27,28]. Recent studies show an interaction between microbiota and TC, which affects thyroid homeostasis and promotes immune escape in cancer. Therefore, in this review, we discuss the relationship between GM and TC and elucidate the mechanisms involved, which may provide potential ideas for the prevention, treatment, and management of TC.

## 2. Imbalance in GM Is Associated with DNA Damage and Immunosuppression

### 2.1. Imbalance of GM Causes DNA Damage

The GM in an imbalanced state leads to a decrease in the abundance of some beneficial bacteria and the overgrowth of other microorganisms, thus leading to an accumulation of endotoxins and exotoxins. These bacterial toxins can directly or indirectly induce DNA damage and genetic instability, which in turn promote tumorigenesis and progression [29,30,31]. A previous study suggests that these dominant microorganisms can interfere with the immune responses and increase local inflammation, thus leading to PIK3CA mutations that may exert cancer-promoting effects in colorectal cancer [32]. Although this mechanism occurs in the gastrointestinal tract, further causing damage to adjacent cells, these stimuli can lead to genetic damage and systemic immune imbalance, resulting in increased secretion of cytokines or chemokines, which may potentially promote tumor development in other organs. If a bacterial infection occurs in the intestine, in addition to the clinical symptoms, such as diarrhea and fever, genotoxins, such as typhoid toxin, cytotoxic distention toxin, and colibactin produced by several Gram-negative bacteria can also cause DNA damage to cells, further resulting in genetic instability [33,34]. New mechanisms through which bacteria and their metabolites or toxins can cause direct DNA damage and oncogenic mutations have been identified. For example, *E. faecalis* infection leads to increased production of hydroxyl radicals, which can cause DNA damage [31]. Similar evidence of DNA damage from a bacterial imbalance in the non-digestive tract has been reported. The relative abundances of *E. coli* and *S. epidermidis* isolated from the peritumor of breast cancer are high and can induce DNA double-strand breaks in HeLa cells [35]. Therefore, dysregulation of the gut microbiota leads to the accumulation of metabolites that, through specific mechanisms, cause DNA damage to the distal glands and continue to cause cancer. In addition, these metabolites can also lead to autoimmune reactions, resulting in an imbalance of endocrine homeostasis, and the occurrence of autoimmune diseases.

### 2.2. Interaction between GM and Immune Regulation

A large microbiota colonizes the gut and may be an important factor in the development of the lymphatic system because 70% of the lymph is distributed in the intestinal mucosa [36]. Microbial disorders can lead to tumor susceptibility by overstimulating CD8(+) T cells and promoting chronic inflammation and early T-cell failure, thereby reducing anti-tumor immunity [37]. Human and mouse studies in tandem verify that the composition of intestinal microbiota gradually changes with increasing age, indicating that the intestinal microbiota of young people and the elderly are significantly different [38,39], which may be one of the reasons why the immune systems of young individuals are better than those of the elderly [40,41]. With the development of genomics and metabolomics, the role of GM in tumorigenesis and therapy is gradually being recognized. In a germ-free mice model, the maturation of immune cells was found to be disturbed due to the lack of microbial stimulation of the immune system [42]. Recent studies show that the microbiome acts as an immune influencing factor to help control the immune system or reduce the occurrence of distant metastases in liver and pancreatic cancers [43,44,45]. Some microorganisms can regulate the immune system of the host to change the tumor microenvironment, thus eventually leading to the immune escape of the tumor, while others can also kill the tumor by activating the immune system [33]. Lactobacillus and Bifidobacterium are common probiotics which have been proven to improve the intestinal environment and exert positive effects on metabolism and immunity [34]. A previous study showed that prebiotics possess anti-tumor immune responses and inhibit tumor growth in a mouse model of melanoma, whereas tumor growth remains unaffected in the control group [46]. Although validated in animal models, it will provide ideas in the study of the effect of prebiotics on the human antitumor immune response. The composition of the gastrointestinal microbiota can impact the immune system. As immunotherapy has gradually become a promising anticancer treatment strategy, its sensitivity and adverse reactions are closely related to GM [33,47]. Whether these can be involved in tumor progression or mitigation through their metabolites is currently a research hotspot.

## 3. GM–Endocrine Homeostasis–Thyroid Axis

### 3.1. GM Affects Endocrine Homeostasis

Iodine deficiency is one of the well-known causes of goiter, which leads to the appearance of thyroid nodules and lays down the groundwork for the development of TC. Iodine is absorbed in the stomach, duodenum, and jejunum, and most of it is transferred to the thyroid gland, where it is concentrated and synthesized into triiodothyronine (T3) and thyroxine (T4). Therefore, the microbiota plays an important role in iodine uptake [12]. In addition, iodine, iron, and copper are essential for the synthesis of thyroid hormones, while selenium and zinc are necessary for the conversion of T4 to T3. Deficiencies of these micronutrients can lead to thyroid malfunction [48]. Therefore, the composition of the GM exerts an impact on the absorption of essential thyroid micronutrients. In an animal study, the binding of bacterial extra-membrane lipopolysaccharide to thyroid cell toll-like receptor 4 (TLR-4) was found to activate the NF-κB signaling pathway, subsequently leading to binding with paired box 8 (PAX8), an important regulator of sodium iodide symporter (NIS) expression. Lactobacillus promotes the uptake of various micronutrients, such as selenium, which increases the binding and activation of PAX8, thereby inducing the transcription of NIS [49]. This shows that homeostasis of the intestinal microbiota influences the function of NIS, thereby further impacting the homeostasis of thyroid functioning. This suggests a relationship of the gut microbiota–endocrine–thyroid axis.

### 3.2. GM and Autoimmune Thyroid Disease

**Figure 1:**
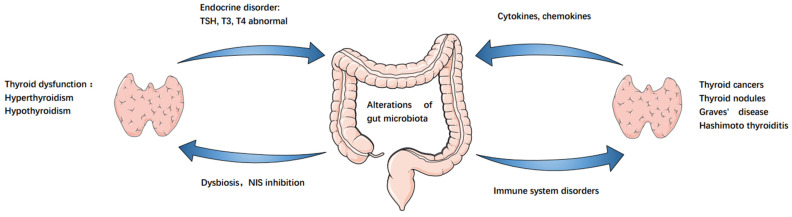
Gut–endocrine–thyroid cancer axis. Alterations of gut microbiota led to NIS inhibition, resulting in decreased thyroid iodine uptake and immune system, resulting in thyroid dysfunction, thyroid autoimmune diseases and thyroid cancer. Similarly, these diseases cause further changes in the gut microbiota.

In addition to this, abnormalities in the intestinal microbiota can cause the development of autoimmune thyroid diseases, resulting in thyroid dysfunction. For example, there is a significant difference in the GM between patients with GD and healthy individuals [12,50]. Whether this difference can intervene in immune modulation of the intestinal tract, thereby leading to the development of GD, is a potential pathogenesis mechanism underlying GD. As compared to controls, the diversity of intestinal microbiota is lower in patients with GD. The abundances of Prevotellaceae and Pasteurellaceae are significantly higher relative to controls, while those of Enterobacteriaceae, Veillonellaceae, and Rikenellaceae are significantly lower [51]. Additionally, two intestinal probiotics, Lactobacillus and Bifidobacterium, reduce HT and GD, and both bacteria negatively correlate with iron and positively with selenium and zinc levels. Therefore, the modulation of the levels of these micronutrients may be a potential strategy for the treatment of these diseases [12]. In contrast, another study demonstrated a significant increase in *Bacillus* spp., along with a decrease in Bifidobacterium spp. in stool samples of patients with HT. In addition, Lactobacilli species are higher in patients who do not take thyroid hormone replacement as compared to those who orally take levothyroxine [52]. There are significant differences in dietary habits between these patients and the healthy control group. Therefore, dietary regulation of microbiota, their metabolites, and direct or indirect effects on intestinal mucosal immune cells directly affect the occurrence of inflammation. The potential microbiota dysbiosis–endocrine homeostasis–thyroid axis schematic is shown in Figure 1.

### 3.3. GM and Thyroid Function Regulation

There is a significant difference in the diversity of GM α and β between patients with hypothyroidism and healthy individuals; stool transplantation from patients with primary hypothyroidism into a mouse model showed a decrease in total thyroxine levels [53]. This demonstrates a strong correlation between thyroid hormone levels and GM. Unfortunately, similar studies have not been confirmed in humans. An analysis of intestinal microflora of hyperthyroidism patients showed that Bifidobacteria and Lactobacillus reduce significantly, while Enterococcus is markedly high in the hyperthyroidism group, confirming the changes in GM characteristics [13]. Similarly, patients with hypothyroidism also face dysbiosis of the GM [53]. This evidence indicates that the thyroid hormone has a certain regulatory effect on GM and can alter the microbiota structure. Postmenopausal women in clinical settings have reduced GM diversity, thus confirming that the diversity of the GM is associated with the metabolism of estrogen [54], a potent growth factor for PTC cells [55], and may serve as potential evidence for the GM–endocrine–thyroid cancer axis.

## 4. Correlation between GM and Cancer

### 4.1. Interrelationship between GM and Gastrointestinal Cancers

The mechanisms underlying tumor formation by the dysbiosis of GM are broadly divided into two categories. The first category includes damage to DNA and its stability, leading to mutations. The second category includes inflammatory responses, such as the activation of Toll-like receptors (TLRs) by tumor-associated microbial communities, thereby stimulating the activation of NF-κB signaling in the tumor microenvironment [36]. In particular, the metabolites of some microbiota strongly influence the immune system’s role in the surveillance of tumor growth.

Some viruses can interact with digestive tumors, such as hepatitis B or C, and esophageal cancer is associated with human papilloma and Epstein–Barr viruses [56,57]. Imbalance in GM first came to the attention of researchers and is the most widely and intensively studied for its role in tumorigenesis and development in the digestive system. Inflammatory bowel disease (IBD) is strongly associated with a high risk of colorectal cancer, and patients with IBD show reduced diversity of intestinal microorganisms as compared to the healthy population, with lower abundances of Firmicutes and Bacteroidetes [58,59]. Moreover, IBD and colorectal cancer have similar pathological and biological processes, as evidenced by increased levels of signaling molecules, such as TNF-a, TGF-β, NF-κB, and ROS, ultimately leading to the dysregulation of intestinal microbial ecology [60]. Some types of colon polyps and advanced adenomas in the colon are also considered precancerous lesions of colon cancer [61,62]. Therefore, researchers have analyzed the differences in microbiota between healthy individuals and patients with colon adenomas. Patients with colon adenomas have significantly lower Bacteroidetes and higher Aspergillus abundance. This imbalance in microflora results in the formation of a biofilm with intestinal mucus; this mucosal microbiota can further induce the development of colon cancer [63].

Some bacteria are known to be involved in tumorigenesis. For example, metabolites and exocytosis of *B. fragilis* can lead to colon tumorigenesis through the upregulation of E-cadherin, β-catenin, NF-κB, and STAT3 [64,65]. Clostridiaceae and Streptococcus exert significant carcinogenic effects on the gastrointestinal tract [66,67]. Helicobacter pylori infection can stimulate immune responses and inflammation, regulate several signaling pathways, and induce gastric acid deficiency, epithelial atrophy, and developmental abnormalities. Therefore, the effective eradication of Helicobacter pylori infection can prevent gastric cancer [68]. Furthermore, Helicobacter pylori infection leads to the methylation of E-cadherin and the CpG islands in tumor-suppressor genes [69,70], which significantly increases the risk of gastric adenocarcinoma. Conversely, some metabolites of GM also exert cancer-suppressive effects. For example, urolithin A, a metabolite found in fruits and nuts, has been shown to inhibit Wnt signaling, resulting in cancer inhibition [71,72].

### 4.2. Interrelationship between GM and Non-Gastrointestinal Cancers

In the non-gastrointestinal tract, the abundance of *E. coli* is significantly higher in patients with liver cirrhosis relative to the healthy population [73]. Therefore, *E. coli* overgrowth in the intestine may be an important factor in the development of hepatocellular cancer. In addition, lipopolysaccharide (LPS) produced by *H. pylori* upregulates the levels of IL-8 and TGF-b1, activates the NF-κB pathway, and promotes the growth and migration of hepatocellular carcinoma cells [74]. In addition, bile acids can regulate the GM. A decrease in bile acid content leads to an imbalance in the intestinal microbiota, thus accelerating inflammation and DNA damage and directly promoting cancer [75,76]. In endocrine organs, such as the pancreas, microorganisms can cause slight or persistent immune and inflammatory reactions, ultimately leading to the formation of pancreatic cancer [77]. Although the evidence is currently scarce, the role of periodontal disease and Streptococcus gingivalis in the etiology of pancreatic cancer also provides new clues for the development of pancreatic cancer [78]. In addition, dysbiosis of the GM may also be a predisposing factor for the development of multiple myeloma [79]. These studies suggest that dysbiosis of the GM not only exerts carcinogenic effects on the digestive system, but also on the non-digestive system.

## 5. Can Dysbiosis of GM Promote the Development of TC?

The role of dysbiosis of the GM in promoting tumors of the digestive system has been confirmed by numerous studies, but validation for extra-intestinal tumors is still needed. However, a study demonstrated the higher microbial richness and significant compositional differences in the GM of TC patients, thus indicating a correlation between GM and TC [48]. However, it is not clear whether the differences in GM lead to the development of TC or whether the development of TC causes a series of endocrine changes, leading to the differences in GM.

Imbalance in the GM can affect the body’s uptake of some micronutrients. Iodine deficiency can lead to goiter or the development of thyroid nodules, thus causing an increased chance of tumorigenesis. In contrast, however, PTC appears to be more common in areas of high iodine intake [80], suggesting a complex relationship between the iodine intake level and TC. Interestingly, GM can affect iodine uptake, NIS, and Tg expression, as well as TSH levels [81,82]. Therefore, GM may be associated with RAI-refractory PTC through a mechanism different from NIS regulation. In addition, selenium affects the composition and colonization of the microbiota in the intestine [83]. There is a correlation between reduced selenium concentration and a higher TC stage [84]. Serum Tg is a sensitive biomarker in patients with PTC, as it can predict distant metastases and identify RAI-refractory PTC [85]. Huang and colleagues showed that when Tg is lowly iodinated, NIS expression and protein kinase A (PKA) activity increase; however, when highly iodinated protein kinase C (PKC) activity increases, NIS expression decreases [86].

In the postoperative management of PTC, radioactive iodine therapy is an important therapeutic method. A previous study showed that NIS plays an important role in mediating the responses to RAI by participating in iodine uptake, the most important rate-limiting step in thyroid hormone production and RAI therapy [87]. GM is one of the important factors affecting iodine uptake, so the alterations of GM will indirectly affect the treatment efficiency of RAI [12]. Similarly, TSH increases iodine uptake by inducing NIS expression and transferring it to the cell membrane. Conversely, high doses of iodine decrease the mRNA and protein expressions of NIS, thus leading to reduced iodine uptake [88], suggesting a potential mechanism underlying resistance to RAI treatment. In addition, the use of iodine-containing contrast agents can have a deleterious effect on the microbiota by binding to the amino acids, tyrosine, and histidine, on bacterial membranes and oxidation cytoplasmic and membrane components [12]. A metabolomic study on metastatic PTC found that diet and GM were strongly associated with metastases. The authors also identified 31 metabolites and that tumor cells promote tumor proliferation through the uptake and synthesis of abundant pre-synthetic biomolecules. Pathway analysis suggested that alanine, aspartate, and glutamate metabolism may be dysregulated, thus leading to metastasis in PTC [89].

Compared to other tumors, TC, a common endocrine tumor, has specific pathogenesis. Most of the above-mentioned studies are based on the evaluation of NIS uptake and iodine metabolism, thus confirming the link between microorganisms and iodine uptake and may provide potential ideas for clinical treatment.

## 6. TC induces Alterations in the Intestinal Microbiota

### 6.1. Thyroid Cancer Triggers Changes in Intestinal Microbiota

Evidence has been found for tumor effects on GM. In recent years, in PTC, some studies on the impact of GM have also gradually emerged. A report characterizing the intestinal microbiota in stool samples from PTC patients and healthy controls showed an increase in the proportion of Firmicutes and Proteobacteria and a decrease in Bacteroidetes in PTC patients. In addition, metabolites in the PTC group are also enriched in pathways related to the tumor, cell growth and necrosis, lipid metabolism, and immune system [90]. Another similar study showed that the microbial changes observed in TC lead to a decrease in aminoacyl- tRNA biosynthesis, homologous recombination, mismatch repair, DNA replication, and nucleotide excision repair. The GM of PTC in N0 and N1 stage subgroups changes significantly [22]. Similarly, in another study, it was confirmed that Sphingomonas is a marker of lymph node metastasis [6]. This suggests that alterations in GM may serve as a potential prognostic indicator for TC. TC can affect the microbial metabolism in the thyroid and intestine, whereby the total number of microorganisms decreases with increasing the distance from the cancer tissue. In addition, the gene sequencing results of tumors from PTC patients show a higher abundance of Proteobacteria, while those of fecal samples show a higher abundance of Firmicutes [91]. These changes are closely related to TSH and T3 levels. The changes in microbiota may affect the TC microenvironment through pyruvate, fatty acid metabolism, glycolysis, or gluconeogenesis. Another analysis of the gut microflora in 74 patients suggests a significantly higher relative abundance of Neisseria and Streptococcus in TC or thyroid nodules as compared to healthy controls. The relative abundances of Butyricimonas and Lactobacillus are significantly lower in the TC and thyroid nodule groups [82]. These findings may provide clues for TC, thyroid nodule, and GM composition. The GM of patients with high-grade thyroid nodules shows higher amino acid degradation and lower butyric acid metabolism. The relative enrichment of L-histidine metabolism pathways is related to the TSH [92]. This may be a potential therapeutic target for GM as a regulator of thyroid metabolism. Serum analysis of distant metastatic PTC in the ablation group suggests that metabolic abnormalities may be associated with different biological behaviors of tumor cells and immune escape; metabolites and gut microbes may play a role in these effects. Alanine, aspartate, glutamate, and inositol phosphate metabolism are the most critical pathways herein [89].

### 6.2. Microbiota Changes in Thyroid Cancer

It is suggested that there are differences in the microbial composition of TC and peritumoral tissues, which can be used as a potential marker to distinguish tumors from normal tissue. In addition to the effects on the microbiota of the digestive tract, 16s rRNA gene sequencing in the thyroid and peritumoral tissues revealed a significant increase in the abundances of Sphingomonas and Aeromonas in tumor tissues, along with a significant increase in the abundances of Comamonas, Acinetobacter, and Peptostreptococcus in peritumoral tissues. A high abundance of Sphingomonas is associated with lymph node metastases [6]. Interestingly, the intra-tumor microbiota also differs according to the tumor subtype and sex. In a microbiological analysis of PTC specimens, an abundant microbiota was found to be highly associated with immune-related genes such as CD4, CCL18 and CXCL14; a significant abundance of microbes in the high-cell subtype and male patient population was associated with higher rates of mutation and inhibition of tumor methylation [93]. This suggests heterogeneity of the tumor and the co-regulation of various hormones and other factors in the abundance and activity of GM. In contrast, Lactobacilli decline significantly in the TC and nodule groups. This genus is an important source of several trace elements in human cells, such as selenium, which has antioxidant and protective effects on the thyroid gland. These alterations in TC and GM are detailed in Table 1.

**Figure 2:**
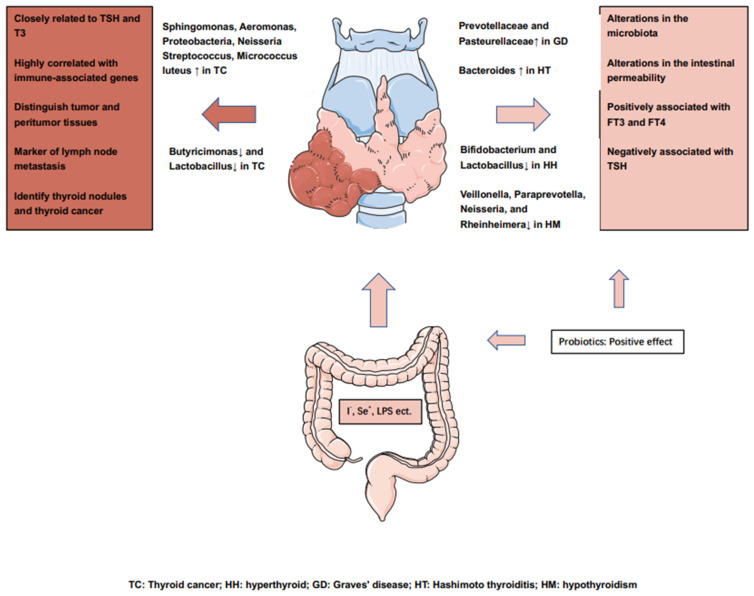
Overview of the influence of the gut microbiota on the thyroid cancers or thyroid diseases.

However, the above studies confirm that the dominant microbiota in thyroid diseases such as hyperthyroidism, hypothyroidism, and HT are different from those in TC, which also suggests that abnormalities in the endocrine system and tumorigenesis affect the GM through different mechanisms. We summarize the influence of GM on thyroid cancer or thyroid disease in Figure 2.

## 7. Future Perspectives

Although it is impossible to prove whether TC affects the composition of the GM or whether the imbalance in GM leads to altered immune functions and ultimately the development of thyroid nodules or formation of TC, the correlation between TC and GM is in the initial stages of research, and more in vivo and clinical studies are still needed to confirm these findings. A healthy diet, with an increased proportion of plant-based foods and a limited proportion of meat, helps establish a healthy GM [94].

In the field of immunotherapy for tumors, improving the GM has become a way to increase the treatment efficacy. Some probiotics can enhance the effect of cancer immunotherapy. Bifidobacterium, a representative probiotic that activates immune system, has been shown to enhance the effects of antitumor drugs in animal studies, although it is still not approved for use in cancer patients [95]. In a mouse model of melanoma, Bifidobacterium enhances the effect of PD-L1 inhibitors, whereby the combination treatment eliminates tumor growth, and enhanced dendritic cell function leads to increased CD8(+) T-cell activity in the tumor microenvironment [96]. Although this has not been proven in humans, GM management combined with cancer immunotherapy may serve as a potential treatment strategy. Lactic acid bacteria HDB1258 isolated from the stool of breastfed infants exert anti-tumor effects by activating innate immunity and enhancing immune responses by increasing natural killer cell cytotoxicity and macrophage phagocytosis, as well as expressions of TNF-α and IL-10 [97]. GM largely influences the sensitivity of tumors to various treatments, especially immunotherapy [98,99,100]. This contributes to the therapeutic activity of CTLA-4 or PD-1/PD-L1-based cancer immunotherapy. The intestinal flora has a modulatory role in immunotherapy for a variety of solid tumors [101], and patients with high Firmicutes and Verrucomicrobia abundances almost universally show higher sensitivity to immune checkpoint inhibitors (ICIs). Unfortunately, TC was not included in this study. Tanoue et al. isolated 11 strains of bacteria from healthy human feces and induced IFN-γ secretion from CD8(+) T cells in mice to enhance the therapeutic effects of ICI [102]. More importantly, GM not only enhances the sensitivity to immunotherapy but also reduces the adverse effects of ICIs [103,104]. Another previous study showed that genetic loading of PD-1 and CTLA-4 antibodies into Salmonella improves the efficiency of drug delivery, thus enabling the use of multiple immunotherapies in combination and improving the treatment efficacy [105]. Moreover, Lactococcus spp. can modulate cellular immunity by maintaining the cytotoxicity of natural killer cells in the body to achieve tumor-killing effects [106,107]. In terms of diagnosis and treatment, Sphingomonas and Aeromonas can distinguish between TC and peritumor tissues, whereby the former is enriched in metastatic lymph nodes. Thus, if we know how to identify these bacteria, it will be of great help for diagnosis and surgery.

## 8. Conclusions

In summary, both the GM and colonized bacteria in TC play a role in maintaining endocrine homeostasis in the thyroid gland and the development of TC. Similar to other cancers, the relationship between TC and intestinal microbiota is interactive and balancing; breaking this balance and forming positive loops between each other through multiple metabolites and pathways can promote tumorigenesis and progression. However, at present, studies are scarce, and it is unclear as to the exact relationship between altered host microbiota, endocrine pathways, and TC. Most of the studies on probiotics are based on animal models, and more in vivo studies are needed to confirm the use of probiotics for TC treatment. Nevertheless, this review is expected to provide new ideas for developing treatment strategies for TC.

## Figures and Tables

**Table 1:** Microbiota differences in thyroid diseases and thyroid cancers.

Samples	Microbes Associated	Roles	Mechanisms	Ref.
TC vs. Peritumor tissue	Sphingomonas and Aeromonas ↑ in TC Comamonas, Acinetobacter, and Peptostreptococcus ↑ in Peritumor Sphingomonas ↑ in N1-stage of TC	Distinguish tumor and peritumor tissues Sphingomonas is a marker of lymph node metastasis	N/A	[6]
HH vs. Healthy control	Bifidobacterium and Lactobacillus↓ in HH Enterococcus ↑ in HH	N/A	N/A	[13]
TC vs. Healthy control	Proteobacteria ↑ in TC	N/A	Decline in aminoacyl—tRNA biosynthesis, homologous recombination, mismatch repair, DNA replication, and nucleotide excision repair	[22]
GD vs. Healthy control	Prevotellaceae and Pasteurellaceae ↑ in GD Enterobacteriaceae, Veillonellaceae and Rikenellaceae↓ in GD	N/A	N/A	[51]
HT vs. Healthy control	Bacteroides ↑ and Bifidobacterium↓ in HT	Zonulin ↑ Alterations in the microbiota and intestinal permeability	N/A	[52]
HM vs. Healthy control	Veillonella, Paraprevotella, Neisseria, and Rheinheimera↓ in HM	Positively associated with FT3 and FT4, and negatively associated with TSH	Increased serum LPS levels	[53]
TC, TN vs. Healthy control	Neisseria ↑ and Streptococcus ↑ in TC and TN Butyricimonas↓ and Lactobacillus↓ in TC and TN	Identify thyroid nodules and thyroid cancer	N/A	[82]
TC vs. Healthy control	Enrichment of 19 and depletion of 8 genera in TC	Lipoprotein A ↑ and apolipoprotein B ↑	Necroptosis Glycerolipid metabolism Fc-gamma R-mediated phagocytosis	[90]
TC vs. Peritumor tissue	Proteobacteria ↑ in TC Firmicutes ↑ in stool of TC	Closely related to TSH and T3	Pyruvate, fatty acid metabolism and glycolysis or gluconeogenesis	[91]
TN vs. Healthy control	Multiple butyrate producing microbes↓	Greater amino acid degradation and lower butyrate production	L-histidine metabolism	[92]
TC in male vs. female	Micrococcus luteus ↑ in TC *Chroococcidiopsis* sp. ↑ in female *M. luteus* and *Bradyrhizobium* sp. BTAi1 ↑ in male	Highly correlated with immune-associated genes Strong positive correlation to MACIS score	DNA checkpoint and damage-related group BRAF V600E mutation p53 instability	[93]

TC: Thyroid cancer; HH: hyperthyroid; GD: Graves’ disease; HT: Hashimoto thyroiditis; HM: hypothyroidism; TN: thyroid nodules.
